# Have Our Attempts to Curb Obesity Done More Harm Than Good?

**DOI:** 10.7759/cureus.10275

**Published:** 2020-09-06

**Authors:** Areeba N Memon, Asavari S Gowda, Bhavana Rallabhandi, Erjola Bidika, Hafsa Fayyaz, Marina Salib, Ivan Cancarevic

**Affiliations:** 1 Internal Medicine, California Institute of Behavioral Neurosciences & Psychology, Fairfield, USA; 2 Neurology, California Institute of Behavioral Neurosciences & Psychology, Fairfield, USA

**Keywords:** obesity, dieting, eating disorders, anorexia nervosa, competent eating

## Abstract

Dieting is a common method for weight loss, maintenance, and prevention of weight gain, but the harmful outcomes of dieting are understudied. Dieting is typically advised for obese patients for the sake of their health, though this does not account for the many complicated factors surrounding obesity. We conducted a search through the PubMed database on obesity, dieting, and eating disorders and did not limit the study by population or year. We found studies showing that although dieting may cause short-term weight loss, it is associated with weight gain in the long-term. We also found studies assessing the negative psychological and physical outcomes of dieting. Though there are many studies that emphasize the negative psychological impact of dieting, few studies have explored how dieting may contribute to the development of eating disorders in the obese. Studies on the physical impact of dieting were less conclusive but warrant further study. While it is difficult to draw any substantial conclusions from the data, our results showed that dieting may carry more risks than benefits as a means to lose weight.

## Introduction and background

“Scholars have become increasingly interested in 'the epidemiology of expertise', recognising that experts play an important role in maintaining the power and legitimacy of polluting industries. Given the enormous political and economic clout of the tobacco industry, it should come as no surprise that experts have been handsomely paid to help the industry emerge victorious...” So reads a historical article by Robert Proctor on the medical community's hand in perpetuating the controversy around smoking [1]. Modern-day health professionals may be shocked to see doctors dismissing the ill effects of smoking when sequelae-like lung cancer are now so well known. However, it showcases that the medical community is capable of pushing incorrect narratives, and may, in fact, do more harm than good when imparting medical advice. While this may be an extreme example, it begs the question: Are there any current medical recommendations that may be harmful to the general population instead of helpful?

Obesity is a disorder of excess body fat, usually defined by a body mass index (BMI) of 30 or more. It is associated with various health problems, such as diabetes mellitus, cardiovascular disease, obstructive sleep apnea, and cancer [2]. A plethora of evidence exists for these complications in current literature, although some studies have highlighted the bias and confounding variables in those studies [3]. Other studies have also found that obesity may not be the harbinger of illness as a popular belief may suggest, though this has also been criticized [4, 5].

Regardless, the fact remains that obesity and its complications are a complex series of processes. Among these, diet and a sedentary lifestyle have been emphasized in particular. Despite interventions to promote weight loss, such as dieting or bariatric surgery, the prevalence of obesity worldwide has only increased [6].

Dieting is a common method for attempting both weight loss and maintenance [7]. Dieting can encompass a variety of food intake changes, but in the case of weight loss, it is usually defined as a restriction in caloric intake. Diets as a method of weight loss or maintenance have also come under scrutiny either because they provide no benefit to overall health or because they lead to long-term weight gain instead [8, 9].

Little emphasis has been made on the harmful effects of dieting, although some have linked its association with eating disorders [10]. Eating disorders are defined by a persistent disturbance of eating or eating-related behavior that impairs physical health or psychosocial functioning. They include binge-eating disorder, anorexia nervosa, and bulimia nervosa [11]. The prevalence of eating disorders varies based on the accuracy of diagnosis and ranges from 8.4%-19.4% in women and 2.2%-13.8% in men [12]. Anorexia nervosa, in particular, has a high mortality rate, with approximately 10% of those affected dying within 10 years of diagnosis [12]. Eating disorders are also associated with a wide range of medical complications, including cardiovascular disease, electrolyte imbalances, and endocrine system disturbances [12].

The lack of literature exploring obesity, dieting, and dieting’s negative consequences is concerning because of the high prevalence of dieting as a mechanism to lose or maintain weight. Both the psychological and physical impact of dieting may be more detrimental to some people than obesity without comorbidities. Patient health is also dependent on their psychological state, and if dieting is truly contributing negatively to it while failing to solve the physical detriments of obesity, dieting should not be recommended.

This paper explores dieting as a solution to obesity and whether dieting comes with more drawbacks than benefits. We begin with whether diets are effective at losing and maintaining weight in the long-term, and then discuss the psychological and physical effects of dieting.

## Review

Methods

We conducted a search through the PubMed database using keywords such as “obesity,” “dieting,” “eating disorders,” and “competent eating” in conjunction. Search results were not limited by population or by year, but greater attention was given to more recent studies, so as to conduct the review with the most up-to-date and relevant studies. Animal studies were included. Reference lists and related articles were also searched for relevant publications. There were no inclusion or exclusion criteria.

Do diets work?

Some controversies exist as to whether diets help patients lose weight [13, 14]. While diets do cause short-term weight loss, substantial evidence exists that diets do not maintain the weight loss or cause weight gain in the long run. Studies have shown that most dieters regain the weight they have lost through dieting, with a longer follow-up period correlating with a larger weight gain, suggesting that diets are ineffective in the long-run. For example, Hensrud et al. followed up on women who had attained normal body weight from dieting and found that after a year, subjects had gained 47% of their weight, while after four years, they regained 87% of it [13]. A long-term randomized study focusing on dieting versus non-dieting control subjects found no statistically significant weight difference in either group. After a two-and-a-half-year follow-up, they found that dieters had kept off an average of only 1.7 kg compared to the control group, concluding that obesity treatment research should focus on better ways to sustain weight loss [14].

Numerous studies have also shown the association between weight loss attempts and future weight gain, even after controlling for confounding variables [15, 16]. Although these studies simply showed an association, Pietiläinen et al. conducted a twin study demonstrating that the association was causal [9]. In monozygotic twins, the co-twin with at least one lifetime intentional weight loss episode was found to be 0.4 kg m^−2^ heavier than their twin who had never dieted, with the same baseline BMI [9]. Although the weight regain is minimal, it is still significant because it indicates that diets, at the very least, do not help maintain weight loss.

This weight gain becomes more apparent in the lean. In the classic Keys et al. starvation experiment, 36 healthy volunteers underwent a four-phase trial: a 12-week control period, a 24 week semi-starvation period that aimed for approximately 25% weight loss, a 12-week rehabilitation period and a final eight-week unrestricted period, where the subjects’ caloric intake was unrestricted but monitored [17]. During the re-feeding period, the subjects became hyperphagic and ate more than their pre-starvation period, leading to weight overshooting. While this study is not considered dieting in the conventional sense, it showcases that any restriction of food can lead to future weight gain.

Overall, these studies demonstrate that dieting is an ineffective method of weight control at best and contributes to weight gain at worst. While it may be easy to dismiss these failures as a personal lack of control, it stands to reason that if the majority of the population is unable to follow through with dieting, then perhaps there is something innately wrong with it. We know that a myriad of factors plays into obesity, such as hormonal disturbances, interactions with the gut microbiome, lack of sleep, and socioeconomic status [18]. Logic would then follow that diets would not be able to address all these factors.

The studies in the lean are relevant because societal influences on people’s perception of what is thin have a significant impact on whether they choose to diet or not [7]. This means that even those considered by medical standards to be underweight or normal weight may choose to diet to obtain an ideal figure, and if it is true that dieting can cause weight gain, especially in thin people, the insistence on diet as a mechanism to maintain, lose or prevent future weight gain could be making the obesity epidemic worse.

Psychological consequences of dieting

Despite the frequency of dieting, little emphasis is given on its psychological impacts on day-to-day life. Numerous studies, however, have shown the negative impacts of food restriction and dieting on psychological functioning [17, 19, 20]. Keys et al. provides data in this regard. The study concluded that prolonged semi-starvation led to pronounced depression, emotional distress, and irritability. As the starvation period wore on, the subjects became increasingly fixated around food, revolving their day around meals, and savoring each meal they were given. More importantly, once the subjects were allowed to eat without inhibition, they reported a loss of control over their desire to gorge on food [17]. While these are the results of starvation, Polivy reviewed these effects on chronic dieters [19]. Remarkably, she found that college students who dieted had similar behaviors to those in the Keys et al. experiment and with patients diagnosed with bulimia nervosa or anorexia nervosa [17, 19]. Her data suggested that chronic dieters may be experiencing psychological deprivation akin to those who starve [19]. Herman et al. assessed subjects’ responses to food. In this study, dieters and non-dieters were given either no prior food or a fattening preload of food before being asked to taste and rate certain foods. They measured how much the subjects ate in order to rate the foods. After a fattening preload, they found that non-dieters would restrict their eating compared to if they had been fed nothing beforehand. This is the normal regulation one would expect in organisms. However, they found that restrained eaters did not do this. When given no prior food, restrained eaters would eat less, but if given a fattening preload, restrained eaters ended up eating more [20]. A few studies have concluded that because dieters ignore natural hunger signals in the body, it leaves them susceptible to other signals that lead to binge eating behaviors [20-22].

**Figure 1:**
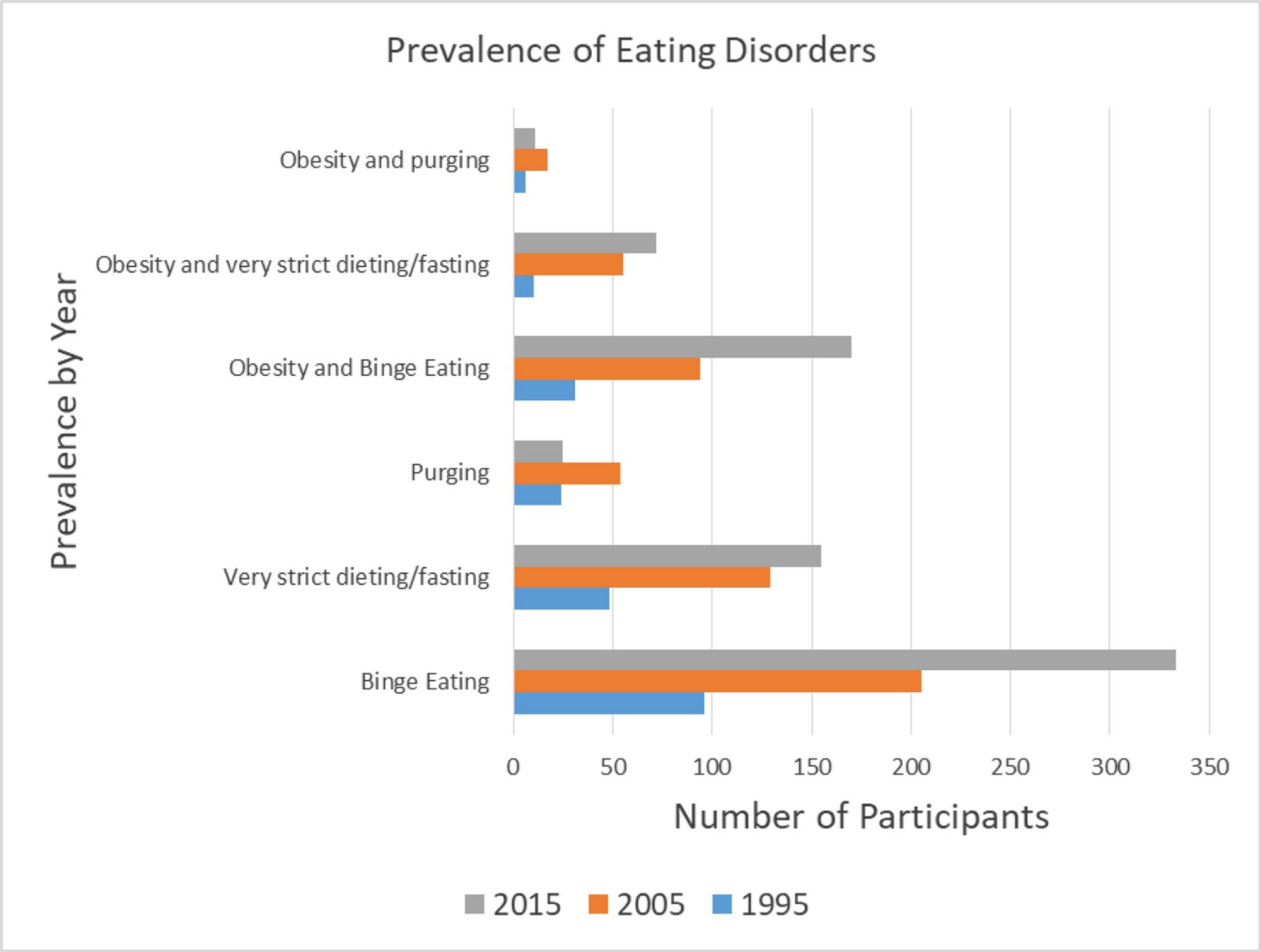
Prevalence of eating disorders by da Luz et al. da Luz et al. [24]

**Figure 2:**
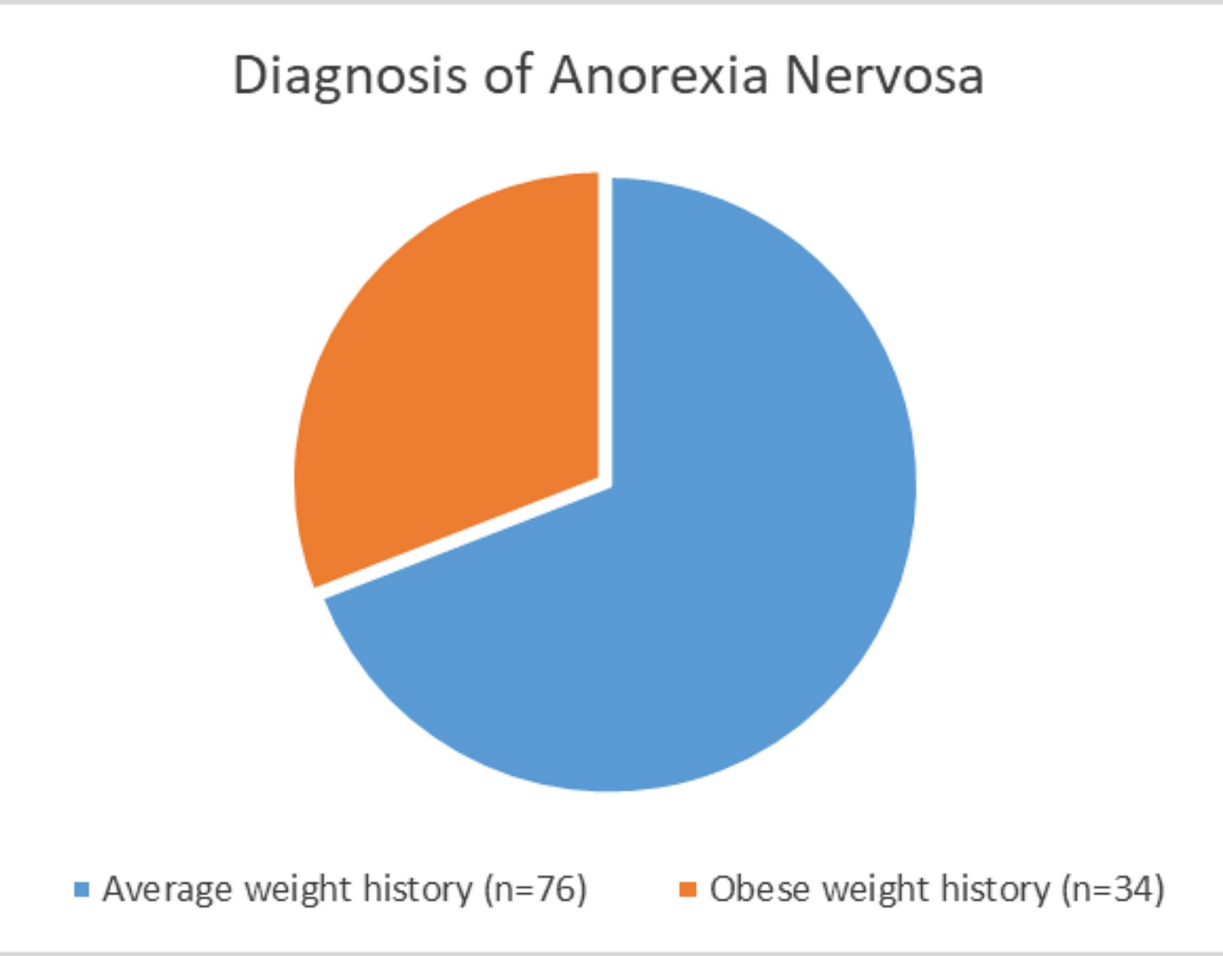
Diagnosis of anorexia nervosa by weight according to Lebow et al. Lebow et al. [25]

There is also significant overlap between obesity and eating disorders. While the prevalence in the general population of eating disorders ranges from 8.4%-19.4% in women and 2.2%-13.8% in men, there is a high co-occurrence of eating disorders with obesity [12, 23, 24]. Eating disorders like anorexia nervosa are also of particular concern because of their high mortality rate [12]. Another study conducted from 2012-2013 of 36,306 people found that those who had met the criteria for the diagnosis of binge eating disorder were more likely to be obese [23]. A study conducted in Australia by da Luz et al. assessing the prevalence of comorbid eating disorders from 1995 to 2015 drew the conclusion that there was a statewide increase in obesity, binge eating and very strict dieting/fasting, and an even greater increase in obesity with comorbid eating disorders [24]. Although the association between obesity and binge eating disorder has been well-established and may seem intuitive, the relationship between obesity and anorexia nervosa has largely been ignored. A diagnosis of anorexia nervosa requires the criterion of a low BMI, which means that obese patients with worrying restrictive eating habits largely go ignored [25]. Lebow et al.’s findings showed that about 36% of adolescents treated for a restrictive eating disorder were previously obese [25]. Summaries of these findings can be found in Figures 1-2.

What concerns us about this association is that dieting and the onset of eating disorders may be related. A population-based cohort study examining adolescents over three years found that female participants who dieted at a severe level were 18 times more likely to develop an eating disorder than those who did not, and female participants who dieted at a moderate level were five times more likely to develop an eating disorder [26]. The study concluded that dieting at an early age was highly predictive of later development of an eating disorder [26]. Similarly, a five-year longitudinal study concluded that dieting and unhealthy weight control behaviors might lead to obesity-related and eating disorder development in adolescents [27]. However, Butryn et al. found that professionally administered weight-loss strategies resulted in minimal risk to overweight and obese adolescents in the development of eating disorders [28]. Additionally, da Luz et al. concluded that, if clinically supervised, dieting did not lead to binge eating behaviors and even decreased binge eating [29].

It is also important to remember that although clinically diagnosed eating disorders are rare, disordered behaviors are incredibly common [30, 31]. Whether disordered behaviors lead to serious eating disorders remains to be explored, but it is crucial to note that this particular phenomenon is understudied.

Interestingly, competent eating has been associated with better health and weight outcomes in patients. Competent eating, as defined by the Satter Eating Competence Model (ecSatter), outlines an approach to eating and food-related attitudes and behaviors that encourage positive bio-psychosocial outcomes [32]. This is done primarily through four domains: eating attitudes, contextual skills, food acceptance, and internal regulation. Essentially, patients are encouraged to enjoy their food, eat to their satisfaction, and have a healthy relationship with their food [32]. Competent eating is associated with better outcomes in individuals, including a lower BMI, lower fasting serum glucose, and lower cardiovascular disease markers such as higher high-density lipoprotein (HDL) cholesterol and lower systolic and diastolic blood pressure [33, 34]. Most importantly, eating competence has a lower association with eating disorders [35, 36].

Given the higher prevalence of obesity and eating disorders, the high prevalence of dieting, and the findings by Herman et al., it would not be a stretch to suggest that there is some relation between dieting and the development of eating disorders [12, 20]. Although Lebow et al. concluded that this was the case, studies on this subject are limited, especially with regards to the development of anorexia nervosa in the obese [25]. While studies have shown an association between dieting and the development of eating disorders in adolescents, few studies have explored this in adults. The studies above suggest that psychological and motivational factors influence this development. Schlundt et al. found that the motivations behind restrictive eating and diagnosed eating disorders were the same [37]. Because eating disorders may be more common than previously thought, and because of the high mortality of eating disorders like anorexia nervosa, more studies need to be conducted on this particular subject, especially on the obese who are largely ignored in studies involving restrictive eating habits. While obesity does have an association with negative health effects, it could be argued that dieting can lead to worse effects, especially if disordered eating behavior is more common than previously thought. This is especially concerning since many studies show that dieting is an ineffective method for weight loss.

Physical consequences

Fewer studies exist on the adverse physical effects of dieting. One study is an observational study assessing weight interventions and their effects on quality of life and mortality. Weight loss was found to have a lower mortality rate in obese people with comorbid conditions but was associated with a higher mortality rate in the healthy obese [38]. Most studies of the physical effects of dieting are usually limited to weight cycling and its relation to the risk of developing diabetes or cardiovascular sequelae. Weight cycling is defined by repeated bouts of weight loss and weight regain. The prevalence of weight cycling has not been well established, but a Finnish study has shown that it is common [39]. A prospective study showed that there were no negative effects of weight cycling on cardiovascular risk factors [40]. A study in Finnish male smokers aged 50-69 years concluded that large weight fluctuations were correlated with diabetes mellitus risk [41]. However, Mackie et al. conducted a systemic literature review that showed no association between weight cycling and future diabetes type 2 risk [42]. Peters et al. showed a decreasing and fluctuating body weight was associated with increased mortality [43]. Another study differentiated from intentional versus unintentional weight loss and drew the conclusion that weight cycling was not associated with higher mortality [44]. One study demonstrated that weight cycling correlated with a lower lean muscle mass, as measured by handgrip strength [45]. By itself, this would not seem to be of particular concern. However, studies have shown that a greater muscle mass is correlated with less risk of developing prediabetes and that sarcopenia is associated with poor glucose metabolism, suggesting that low muscle mass could be predictive of the development of diabetes mellitus [46, 47].

The mechanism for these physical consequences has also been studied. Several hormonal adaptations have been identified that contribute to weight regain after bouts of weight loss. The most pertinent of which is leptin. Leptin is an important regulator of satiety and weight gain. Although leptin has been shown to predict weight regain in mice, this has not been the case in humans [48]. Studies have shown that leptin levels fall due to fasting and can lead to changes in energy balance [48]. These low leptin levels can then lead to overfeeding and suppresses energy expenditure [48]. Strohacker et al. concluded that there was no consistent relationship between leptin sensitivity during weight loss and later weight regain [49]. Although leptin has not been shown to lead to weight gain, it has been shown to affect behaviors. Farr et al. did find through neuroimaging that although leptin replacement in hypoleptinemic women did not alter brain structure, it altered feeding and reward-related behaviors, which could affect weight regain [50].

Overall, it is difficult to conclude any substantial physical health risk associated with dieting. Studies on hormonal changes have demonstrated no physical changes to health from dieting. Weight cycling, in particular, does not seem to show any increased health risks, though the results are mixed. Most relevant is Bosomworth’s study showing that mortality increases with dieting in the healthy obese [38]. This would indicate that although obesity is associated with a higher morbidity and mortality rate, the obese who do not suffer from any comorbid conditions might be harmed by attempts to diet.

What this means

Given the vastness of this data, the most pertinent question to be raised after all this discussion is: despite so much contradictory evidence with regards to obesity and dieting, why do we continue to recommend dieting to patients? Perhaps in an effort to combat the obesity epidemic, we have continually chased some solution to give to our patients, even if those efforts have not been successful. Perhaps the idea that fat is undesirable has been so deeply drilled into our minds as doctors, that it is challenging to consider that something else could be worse. However, we must be honest with ourselves and with our patients. Many factors that affect obesity lie outside our control, and it is better to have a fat and happy patient than a skinny, miserable, and dead one. We recommend approaching obese patients holistically and with compassion. It can be difficult for patients to lose weight, so we recommend encouraging patients to adopt healthy attitudes towards eating instead of chasing after weight loss. 

Limitations

It is difficult to account for obesity alone when so many diseases overlap with it. As some of the cited studies have pointed out, it is common for confounding variables to exist in such studies.

Certain terms, such as dieting, weight cycling, and competent eating, are not well defined. For example, weight cycling can include small or large differences in weight, and there is no defined period of time over which this repeated loss and regain can occur.

It is also difficult to establish a cause-and-effect relationship with many of these studies since most of them only demonstrate an association.

No quality appraisal was done.

## Conclusions

Dieting is a common method for weight control and may carry more risks to health than benefits. Studies have shown that food restriction is a poor mechanism for weight loss and may instead contribute to weight gain. Dieting may also lead to the development of eating disorders, which can be much more deleterious to health than obesity. While the physical consequences are not as extreme, studies show some credibility in the idea that dieting could lead to adverse outcomes in certain populations. If dieting does not help patients lose weight and leads to psychological and physical adverse effects, then the medical community should review it as a recommendation to attain better health. Physicians should emphasize overall healthy habits in patients without focusing strictly on weight loss. Regardless of these findings, the fact remains that dieting clearly has not led to a solution to the epidemic. More studies should be done focusing on the health risks of dieting, especially in the obese who are encouraged to diet for health reasons.

## References

[1] Proctor RN (2006). "Everyone knew but no one had proof": tobacco industry use of medical history expertise in US courts, 1990-2002. Tob Control.

[2] Pi-Sunyer X (2009). The medical risks of obesity. Postgrad Med.

[3] Franks PW, Atabaki-Pasdar N (2017). Causal inference in obesity research. J Intern Med.

[4] Amundson DE, Djurkovic S, Matwiyoff GN (2010). The obesity paradox. Crit Care Clin.

[5] Chrysant SG, Chrysant GS (2019). The single use of body mass index for the obesity paradox is misleading and should be used in conjunction with other obesity indices. Postgrad Med.

[6] (2020). Prevalence of obesity and severe obesity among adults: United States, 2017-2018. https://www.cdc.gov/nchs/products/databriefs/db360.htm.

[7] Santos I, Sniehotta FF, Marques MM, Carraça EV, Teixeira PJ (2017). Prevalence of personal weight control attempts in adults: a systematic review and meta-analysis. Obes Rev.

[8] Mann T, Tomiyama AJ, Westling E, Lew AM, Samuels B, Chatman J (2007). Medicare's search for effective obesity treatments: diets are not the answer. Am Psychol.

[9] Pietiläinen KH, Saarni SE, Kaprio J, Rissanen A (2012). Does dieting make you fat? a twin study. Int J Obes.

[10] Hay P, Mitchison D (2019). Eating disorders and obesity: the challenge for our times. Nutrients.

[11] American Psychiatric Association (2013). Diagnostic and Statistical Manual of Mental Disorders, Fifth Edition.

[12] Galmiche M, Déchelotte P, Lambert G, Tavolacci MP (2019). Prevalence of eating disorders over the 2000-2018 period: a systematic literature review. Am J Clin Nutr.

[13] Hensrud DD, Weinsier RL, Darnell BE, Hunter GR (1994). A prospective study of weight maintenance in obese subjects reduced to normal body weight without weight-loss training. Am J Clin Nutr.

[14] Jeffery RW, Wing RR (1995). Long-term effects of interventions for weight loss using food provision and monetary incentives. J Consult Clin Psychol.

[15] Kroke A, Liese AD, Schulz M, Bergmann MM, Klipstein-Grobusch K, Hoffmann K, Boeing H (2002). Recent weight changes and weight cycling as predictors of subsequent two year weight change in a middle-aged cohort. Int J Obes.

[16] Field AE, Aneja P, Austin SB, Shrier LA, de MC, Gordon-Larsen P (2007). Race and gender differences in the association of dieting and gains in BMI among young adults. Obesity.

[17] Keys A, Brozek J, Henschel A (1950). The biology of human starvation: volume II. http://www.jstor.org/stable/10.5749/j.cttttqzj.

[18] Hruby A, Hu FB (2015). The epidemiology of obesity: a big picture. Pharmacoecon.

[19] Polivy J (1996). Psychological consequences of food restriction. J Am Diet Assoc.

[20] Herman CP, Polivy J, Esses VM (1987). The illusion of counter-regulation. Appetite.

[21] Polivy J, Herman CP (1985). Dieting and binging: a causal analysis. Am Psychol.

[22] Knight LJ, Boland FJ (1989). Restrained eating: an experimental disentanglement of the disinhibiting variables of perceived calories and food type. J Abnorm Psychol.

[23] Udo T, Grilo CM (2018). Prevalence and correlates of DSM-5 eating disorders in nationally representative sample of united states adults. Biol Psychiatry.

[24] da Luz FQ, Sainsbury A, Mannan H, Touyz S, Mitchison D, Hay P (2017). Prevalence of obesity and comorbid eating disorder behaviors in South Australia from 1995 to 2015. Int J Obes.

[25] Lebow J, Sim LA, Kransdorf LN (2015). Prevalence of a history of overweight and obesity in adolescents with restrictive eating disorders. J Adolesc Health.

[26] Patton GC, Selzer R, Coffey C, Carlin JB, Wolfe R (1999). Onset of adolescent eating disorders: population based cohort study over 3 years. BMJ.

[27] Neumark-Sztainer D, Wall M, Guo J, Story M, Haines J, Eisenberg M (2006). Obesity, disordered eating, and eating disorders in a longitudinal study of adolescents: how do dieters fare 5 years later?. J Am Diet Assoc.

[28] Butryn ML, Wadden TA (2005). Treatment of overweight in children and adolescents: does dieting increase the risk of eating disorders?. Int J Eat Disord.

[29] da Luz FQ, Hay P, Gibson AA (2015). Does severe dietary energy restriction increase binge eating in overweight or obese individuals? a systematic review. Obes Rev.

[30] Mond J, Hall A, Bentley C, Harrison C, Gratwick-Sarll K, Lewis V (2014). Eating-disordered behavior in adolescent boys: eating disorder examination questionnaire norms. Int J Eat Disord.

[31] Kaltiala-Heino R, Rissanen A, Rimpelä M, Rantanen P (1999). Bulimia and bulimic behaviour in middle adolescence: more common than thought?. Acta Psychiatr Scand.

[32] Satter E (2007). Eating competence: definition and evidence for the Satter Eating Competence model. J Nutr Educ Behav.

[33] Quick V, Shoff S, Lohse B, White A, Horacek T, Greene G (2015). Relationships of eating competence, sleep behaviors and quality, and overweight status among college students. Eat Behav.

[34] Lohse B, Psota T, Estruch R (2010). Eating competence of elderly Spanish adults is associated with a healthy diet and a favorable cardiovascular disease risk profile. J Nutr.

[35] Stotts Krall J, Lohse B (2009). Interviews with low-income Pennsylvanians verify a need to enhance eating competence. J Am Diet Assoc.

[36] Lohse B, Satter E, Horacek T, Gebreselassie T, Oakland MJ (2007). Measuring eating competence: psychometric properties and validity of the ecSatter Inventory. J Nutr Educ Behav.

[37] Schlundt DG, Johnson WG (1990). Eating disorders: assessment and treatment. https://psycnet.apa.org/record/1990-97518-000.

[38] Bosomworth NJ (2012). The downside of weight loss: realistic intervention in body-weight trajectory. Can Fam Physician.

[39] Lahti-Koski M, Männistö S, Pietinen P, Vartiainen E (2005). Prevalence of weight cycling and its relation to health indicators in Finland. Obes Res.

[40] Wing RR, Jeffery RW, Hellerstedt WL (1995). A prospective study of effects of weight cycling on cardiovascular risk factors. Arch Intern Med.

[41] Kataja-Tuomola M, Sundell J, Männistö S, Virtanen MJ, Kontto J, Albanes D, Virtamo J (2010). Short-term weight change and fluctuation as risk factors for type 2 diabetes in Finnish male smokers. Eur J Epidemiol.

[42] Mackie GM, Samocha-Bonet D, Tam CS (2017). Does weight cycling promote obesity and metabolic risk factors?. Obes Res Clin Pract.

[43] Peters ET, Seidell JC, Menotti A (1995). Changes in body weight in relation to mortality in 6441 European middle-aged men: the Seven Countries Study. Int J Obes Relat Metab Disord.

[44] Stevens VL, Jacobs EJ, Sun J (2012). Weight cycling and mortality in a large prospective US study. Am J Epidemiol.

[45] Rossi AP, Rubele S, Calugi S (2019). Weight cycling as a risk factor for low muscle mass and strength in a population of males and females with obesity. Obesity.

[46] Srikanthan P, Karlamangla AS (2011). Relative muscle mass is inversely associated with insulin resistance and prediabetes: findings from the third National Health and Nutrition Examination Survey. J Clin Endocrinol Metab.

[47] Srikanthan P, Hevener AL, Karlamangla AS (2010). Sarcopenia exacerbates obesity-associated insulin resistance and dysglycemia: findings from the National Health and Nutrition Examination Survey III. PLoS One.

[48] Ahima RS (2008). Revisiting leptin's role in obesity and weight loss. J Clin Invest.

[49] Strohacker K, McCaffery JM, MacLean PS, Wing RR (2014). Adaptations of leptin, ghrelin or insulin during weight loss as predictors of weight regain: a review of current literature. Int J Obes.

[50] Farr OM, Fiorenza C, Papageorgiou P (2014). Leptin therapy alters appetite and neural responses to food stimuli in brain areas of leptin-sensitive subjects without altering brain structure. J Clin Endocrinol Metab.

